# Clinical safety and outcome of recombinant tissue plasminogen activator in patients with stroke attributable to small artery occlusion: A protocol for systematic review and meta-analysis

**DOI:** 10.1097/MD.0000000000026453

**Published:** 2021-06-25

**Authors:** Li-Yan Ni, Ji-You Tang

**Affiliations:** a Department of Neurology, Shandong Provincial Qianfoshan Hospital, Shandong University; b Department of Neurology, Juye County People's Hospital, Shandong, People's Republic of China.

**Keywords:** protocol, recombinant tissue plasminogen activator, small artery occlusion, stroke, thrombolysis

## Abstract

**Background:**

Recent observations raised concern that the intravenous recombinant tissue plasminogen activator (rt-PA) may result in damage to stroke patients caused by small artery occlusion (SAO). Thus, we perform a protocol for meta-analysis to investigate the efficacy and safety of intravenous thrombolysis with rt-PA in SAO-patients.

**Methods:**

The search-style electronic libraries, including Pubmed, Embase, the Cochrane Library, Web of Science, Wanfang Data, VIP Chinese Journals, and China Biomedical Literature Service System are used for document retrieval in June 2021 with no restrictions on language. The risk of bias in include articles will be assessed using the Cochrane Risk of Bias Tool. We perform the meta-analysis by Stata version 10.0 software and calculated the statistics using the inverse variance statistical method. Binary outcomes are presented as Mantel-Haenszel-style risk ratios with 95% confidence interval. Continuous outcomes are reported as mean differences.

**Results:**

The results of the article will be shown in a peer-reviewed journal.

**Conclusion:**

Intravenous rt-PA may be effective and safe in SAO-patients.

## 1 Introduction

Stroke is the second most prevalent cause of death in the world and the major cause of adult disability.^[1–3]^ As increase of life expectancy, the burden of stroke increases worldwide, particularly in middle- and low-income countries.^[4,5]^ Stroke seriously affects people's lives, health, and brings heavy material, economic, and mental burden to society, families and patients.^[6]^

To reduce the burden associated with stroke, investigations of efficacy of available treatments for patients with stroke such as intravenous injection of recombinant tissue plasminogen activator (rt-PA) are necessary.^[7,8]^ Since the US Food and Drug Administration approval in 1996, intravenous thrombolysis (IVT) with rt-PA is the only effective and recognized method in the treatment of acute stroke. The response of treatment seems to be independent of underlying stroke mechanisms. Whether this is also true for stroke attributable to small artery occlusion (SAO) is under debate, because the presumed mechanism of SAO suggests that this stroke subtype may not respond to IVT.

In the placebo-controlled randomized IVT trial, few patients suffered from stroke due to SAO, in spite of this cause accounted for 16% to 23% of all the strokes.^[9]^ In addition, patients with lacunar stroke treated by IVT have a poorer functional prognosis and higher risk of early deterioration than non-lacunar stroke patients treated by IVT.^[10]^ Moreover, leukoaraiosis, which occurs frequently in patients with SAO, has been confirmed to be a risk factor for the symptomatic intracranial hemorrhage after IVT.^[11]^ Thus, the bleeding risk related to IVT may be higher in SAO-patients when compared to patients with other stroke etiologies other than SAO. Recent observations raised concern that the use of rt-PA might cause harm in patients with strokes attributable to SAO. Thus, we conduct a protocol for systematic review and meta-analysis to investigate the efficacy and safety of intravenous IVT with rt-PA in SAO-patients.

## 2 Methods

This meta-analysis was registered at Open Science Framework registries (registration number: 10.17605/OSF.IO/ES39Z) and was conducted in accordance with the guidelines of the Preferred Reporting Items for Systematic Reviews and Meta-Analyses Protocols statement guidelines.^[12]^ Ethics application was not required as this study is based on published trials.

### 2.1 Search strategy

The search-style electronic libraries, including Pubmed, Embase, the Cochrane Library, Web of Science, Wanfang Data, VIP Chinese Journals, and China Biomedical Literature Service System are used for document retrieval in June 2021 with no restrictions on language. The search terms include “recombinant tissue plasminogen activator OR rt-PA,” “stroke OR cerebral infarction OR cerebral ischemia,” and “small artery occlusion.” These terms are searched in the combination of Medical Subject Headings (MeSH) and text words in the title and abstract. References to relevant review articles will be manually searched to identify additional studies

### 2.2 Inclusion and exclusion criteria

Studies are considered potentially eligible for this systematic review if they meet the following inclusion criteria: the objects were definitely diagnosed with stroke attributable to SAO; the intervention group was treated with intravenous rt-PA, while the control group was given placebo or conventional treatment; main outcome and complication measures were independence (modified Rankin scale ≤2) at 3 months, intracranial hemorrhage, and recurrent ischemic stroke; randomized controlled trials. Studies would be excluded if they were available as case reports, letters, biochemical trials, conference abstracts, and reviews or if predefined outcome data required for analyses were lacking.

### 2.3 Data extraction

Two independent authors will extract the below descriptive information from the included articles: demographic information of patients, such as average age, number of patients, sex ratio and body mass index; study characteristics, such as author, year of publication, study language, study design, and the average follow-up period, and details of interventions and outcome measures. If the data cannot be directly extracted or is missing, we will contact the relevant author to ensure that the information is complete. Otherwise, we will calculate them with the guideline of Cochrane Handbook for Systematic Reviews of Interventions 5.1.0.

### 2.4 Quality evaluation

We use the Cochrane Collaborations risk of bias tool to assess the methodological quality and risk of bias of identified studies. Seven specific domains are evaluated in this tool: sequence generation, allocation concealment, blinding of participants and personnel, blinding of outcome assessment, incomplete outcome data, selective outcome reporting, and other issues. Each domain could be assigned as low risk of bias, high risk of bias, or unclear risk of bias based on the judgment criteria. To evaluate publication bias, we perform a funnel plot if the number of included studies is sufficient (>10 articles). A symmetrical funnel plot indicates no possibility of publication bias, whereas an asymmetrical funnel plot indicates a high possibility of publication bias. If we identify publication bias through analysis of the funnel plot, we may discuss possible reasons such as small-study effects.

### 2.5 Statistical analysis

We perform the meta-analysis by Stata version 10.0 software and calculate the statistics using the inverse variance statistical method. Binary outcomes are expressed as Mantel-Haenszel-style risk ratios with 95% confidence intervals. Continuous outcomes are presented as weighted mean differences (WMDs). Heterogeneity among the studies is quantified with the *I*^2^ statistic. A random-effect model is adopted when *I*^2^ > 50% or *P* < .1; otherwise, heterogeneity is negligible and a fixed-effect model is used.

## 3 Discussion

To the best of our knowledge, this is the initial meta-analysis to investigate the efficacy and safety of intravenous IVT with rt-PA in SAO-patients. Ischemic stroke is characterized by highly differentiated subtypes.^[13]^ SAO is a major ischemic stroke subtype that occurs more commonly in Asian than Western populations.^[14]^ However, to date, no evidence has shown the effectives of intravenous rt-PA for patients with stroke attributable to SAO. We acknowledge some of the limitations of this study. First, few studies have reported relevant topic and the sample size may be small; secondly, sometimes it is hard to distinguish different subtypes of ischemic stroke, which may affect the results of this study; thirdly, due to the low quality of the included studies, the evidence level may be declined. To draw more robust evidence, further randomized controlled trials are required.

## Author contributions

**Conceptualization:** Li-Yan Ni.

**Formal analysis:** Li-Yan Ni.

**Funding acquisition:** Ji-You Tang.

**Investigation:** Li-Yan Ni

**Methodology:** Ji-You Tang.

**Study design:** Li-Yan Ni

**Writing – original draft:** Li-Yan Ni

**Writing – review & editing:** Ji-You Tang.
